# Evaluation of anticancer effects of frankincense on breast cancer stem‐like cells

**DOI:** 10.1002/cnr2.1693

**Published:** 2022-08-08

**Authors:** Mohammad Kamalabadi Farahani, Fateme Sadat Bitaraf, Amir Atashi, Zahra Jabbarpour

**Affiliations:** ^1^ Department of Tissue Engineering, School of Medicine Shahroud University of Medical Sciences Shahroud Iran; ^2^ Department of Medical Biotechnology, School of Medicine Shahroud University of Medical Sciences Shahroud Iran; ^3^ Department of Medical Laboratory Sciences, School of Paramedical Shahroud University of Medical Sciences Shahroud Iran; ^4^ Gene Therapy Research Center, Digestive Disease Research Institute Tehran University of Medical Sciences Tehran Iran

**Keywords:** cancer stem cells, frankincense, sphere formation, triple negative breast cancer

## Abstract

**Background:**

Relapse and metastasis in breast cancer are linked to cancer stem cells (CSCs) resistant to anticancer therapies. The presence of cancer stem‐like cells (CSLCs) and their ability to self‐renew is determined by in vitro spheroid formation.

**Aims:**

Many studies have found that frankincense has anticancer impacts, although these effects on breast CSLCs have never been evaluated.

**Methods and results:**

A population of heterogeneous breast tumor cells was extracted from the tumor mass after generating an animal model of triple‐negative breast cancer (TNBC). Spheroid formation was used as an in vitro assay to determine the existence of CSLCs in these cells. MTT assay was used to determine frankincense's cytotoxic activity. An annexin V‐ propidium iodide (PI) staining and scratch test were used to assess the induction of apoptosis and antimetastatic effects of frankincense. The frankincense extract has significant cytotoxic and apoptotic effects on breast CSLCs. Although, the breast CSLCs are more resistant to these impacts than other breast cancer cells.

**Conclusion:**

Our study is the first report that indicates that frankincense extract has anticancer properties in breast CSLCs. Compared to many anticancer chemicals, which have limited potential to battle cancer stem cells, frankincense is an appropriate option to combat breast CSCs.

## 1 INTRODUCTION

The most frequent malignancy among women worldwide is breast carcinoma.^1^ Triple‐negative breast cancer (TNBC) is the most aggressive and invasive type, with a dismal prognosis among all types of breast cancer. Patients with TNBC are currently treated with chemotherapeutic drugs.^2^ For some kinds of breast cancer, innovative tailored medicines are improving patient outcomes. However, in the case of TNBC, this sort of treatment poses significant and fundamental difficulties. Chemoresistance, recurrence, and metastasis are the most common complications among TNBC patients.^3^

The presence of a small minority of stem cells within heterogeneous populations of cancer cells has been explained by decades of cancer cell research. Cancer stem cells (CSCs) also known as cancer‐initiating cells, are special cells that initiate cancer. Surface indicators, multidrug resistance pumps, and altered self‐renewal pathways differentiate these cells. They have an essential role in cancer carcinogenesis and metastasis.^4^ Several investigations in breast cancer have revealed the existence of breast CSCs. They stressed the importance of these cells in tumor development, metastasis, and resistance to current cancer treatments.^5^ CSCs have been shown to form multicellular spheroid forms when cultivated under specific conditions.^6^ These sphere‐forming cells, which allow CSCs to proliferate and multiply, are used as a standard experimental test for determining the potential of stemness in cancer cells and are the greatest instrument for determining cancer stemness.^7^

Traditional medicine products, which have been used for many years, are still popular among the majority of people around the world. These items are usually affordable and have no adverse side effects. Frankincense, an aromatic resin derived from Boswellia trees (Burseraceae family), is used to treat various ailments, including cancer.^8^ Boswellic acid, as the primary component of frankincense, has been found to induce apoptosis in different cancer cells, including prostate,^9^ colon,^10^ melanoma,^11^ hepatocellular,^12^ leukemia, and brain.^13^

Several authors have underlined the anticancer capabilities of frankincense in preclinical and clinical trials in breast cancer,^14^, ^15^, ^16^, ^17^ but these effects on breast CSCs have not been previously documented. The goal of this study was to assess these effects.

## 2 MATERIALS AND METHODS

### 2.1 Cell culture

The murine mammary cancer cell line 4T1 was obtained from the Pasteur Institute of Iran's cell bank (C604) and cultured in high glucose cell culture media [DMEM (Dulbecco's Modified Eagle's Medium) with 10% FBS and 2% Penicillin–Streptomycin (both from Gibco, USA)]. The cells were incubated at 37°C with 95% air and 5% carbon dioxide (CO_2_).

### 2.2 Breast tumor induction and isolation of heterogeneous population of cancer cells

Induction of mammary tumors was performed as described in our previous study.^18^ Briefly, female BALB/c mice weighing 20–25 g were obtained from the Royan Institute (Iran). The animals were kept in cages at 12 h photoperiod while they had free access to food and water. The ethics committee of Shahroud University of Medical Sciences approved this study for ethics in animal research (registration number: IR.SHMU.REC.1398.109). Subcutaneous injection of 4T1 cells (1 × 10^5^) was performed into the mice's flank (or the right hind limb). For isolation of a heterogeneous population of tumor cells, as described in our previous work,^19^ tumors were excised from mice after 35 days of tumor induction. Mincing and enzymatical digestion of tumor tissue were performed in aseptic conditions. The digested tumor was filtered through 70 μm cell strainers and cultured in a DMEM with 10% FBS and 2% Penicillin–Streptomycin (all from Gibco, USA). The cells were ultimately incubated at 37°C in 5% CO_2_ and passaged twice.

### 2.3 Preparation of frankincense extract

The dried powder of gum resin (5 g) from Boswellia sacra (gifted from Dr. Fatemeh Jamshidi‐Addgene‐University of Nizwa‐Oman) was extracted in 50 ml of methanol in a mechanical shaker at room temperature for 48 h. The mixture was then filtered with Whatman no. 1 filter paper. The filtrates obtained from methanol extraction were evaporated to dryness at 45°C in an oven. The dried extract sample was dissolved in sterile dimethyl sulfoxide (DMSO) and stored at −20°C until use.

### 2.4 Cytotoxic effect of frankincense extract on breast cancer spheroids

Suspension and culture of a heterogeneous population of cancer cells (isolated in earlier procedures) were produced as follows:

1. For two‐dimensional (2D) cells were seeded in 96‐well culture plates at a density of 1 × 10^4^ cells/well and cultivated as stated previously. The cell culture media was replaced after 24 h with a complete medium supplemented with various extract concentrations (20, 40, 80, 100, 150, and 200 μg/ml). After 48 h, the medium was withdrawn, and 50 microliters (μl) of MTT solution (5 mg/ml) (Sigma) were added to the cultures. Incubation was maintained for another 4 h, after which 150 μl of DMSO was added. After allowing the formed formazan crystals to dissolve for 30 min, the optical density at 570 nm was measured using a CYTATION/5 image reader (Bio‐Tek Instrument, USA). Finally, cell viability was calculated as a percentage of control wells using the formula:
$$\text{Cell viability}\;\left(\%\right)=\frac{\left(\text{absorbance of treated well}\right)}{\left(\text{absorbance of control well}\right)}\times 100$$
  The control in this equation refers to a culture medium containing cells. This experiment was carried out three times.
2. To make 3D spheroids, cells were seeded at a density of 1 × 10^4^ cells per well in a 96‐well ultra‐low‐attachment microplate and cultured in spheroid forming media made up of high‐glucose DMEM with 0.5% FBS and 2% Penicillin–Streptomycin (all from Gibco, USA) at 37°C in a humidified atmosphere of 5% CO_2_. The tumor spheroids were then treated for 48 h with various doses of extract (20, 40, 80, 100, 150, and 200 μg/ml), and an MTT test was carried out as described.

### 2.5 Apoptosis assay

For the apoptosis assay, 2D monolayer and 3D spheroids of breast cancer cells were produced and treated for 48 h with the extract (105 μg/ml). MabTag's Annexin‐V apoptosis detection kit (Biolegend, Cat No. 640914) was used to perform cell apoptosis assays according to the manufacturer's protocol. Flow cytometric analysis was performed with FlowJo software, version 7.2.2.

### 2.6 Wound healing and invasion assays

1 × 10^6^ cells were seeded in each well of 12 well plates and cultured for 24 h. Subsequently, cells were maintained in a culture medium without FBS for 16–24 h. In the next step, the cell monolayer was scratched with a 200‐μl pipette tip to create a wound. In the final step, the cells were maintained with or without extract (105 μg/ml) for 24 h in a serum‐containing medium, and cells migrating from the leading edge were photographed at 0 and 24 h using a CK40 inverted microscope (Olympus, Tokyo, Japan).

### 2.7 Statistical analysis

All experiments were performed in triplicate. The mean and standard deviation are used to express all of the findings. For data analysis, we utilized Graphpad prism version 6.0 and the paired samples *t* test. It was determined that *p* < .05 was statistically significant.

## 3 RESULTS

### 3.1 Isolation of heterogeneous population of tumor cells

**FIGURE 1:**
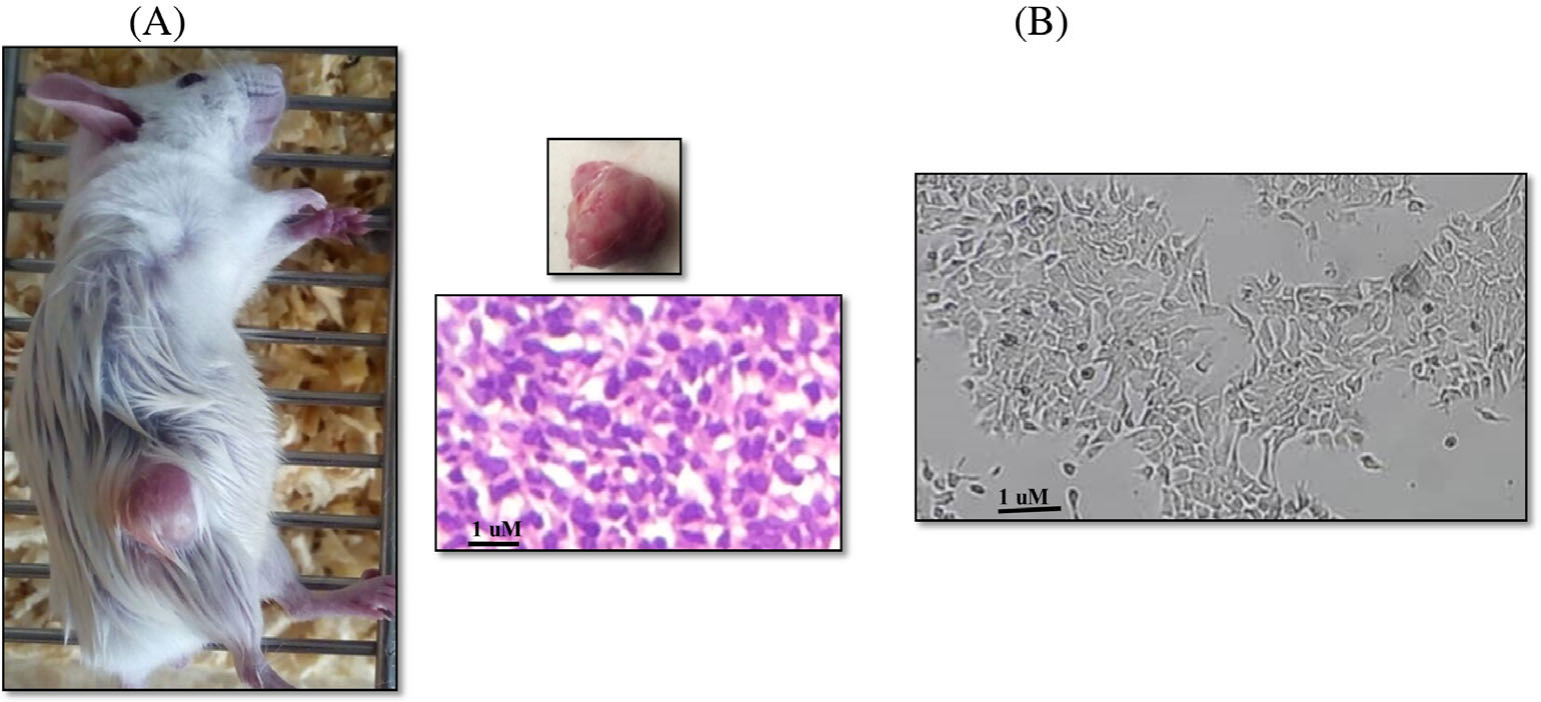
Preparation of heterogeneous population of tumor cells. (A) After 20 days of tumor induction in Balb/c mice, a palpable tumor mass was generated. (B) After tumor isolation and pathological confirmation, the heterogeneous population of tumor cells was isolated on tumor tissues.

Due to many passages and modifications, most breast cancer cell lines have changed their function and genome. Therefore, we decided to make spheroids from a heterogeneous population of primary tumor cells. TNBC, an animal model of breast cancer, was created for this purpose (Figure 1A). When the tumor mass was palpable (20 days after tumor induction in BALB/c mice), it was aseptically excised (Figure 1B). On tumor samples, H and E staining and pathological confirmation were performed (Figure 1B). Enzymatic and mechanical digestion was used to separate the diverse population of tumor cells from the tumor mass (Figure 1B).

### 3.2 Multicellular breast cancer spheroids formation

**FIGURE 2:**
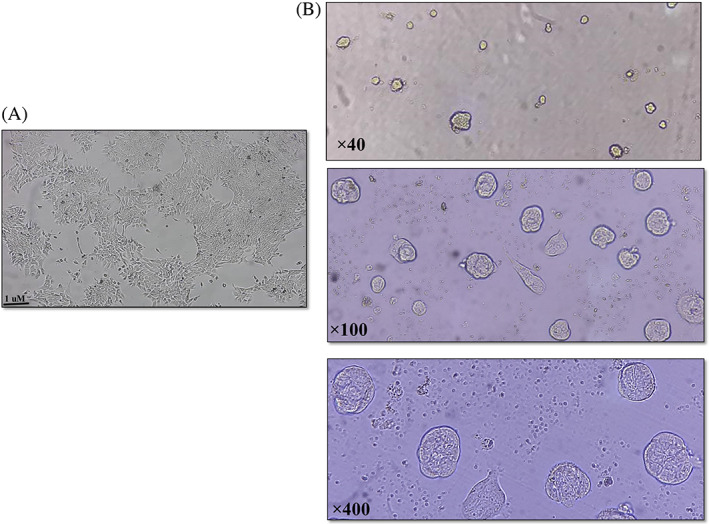
Spheroid conduction in heterogeneous population of tumor cells. (A) 2D cell culture of a heterogeneous population of tumor cells isolated from the primary tumor mass. (B) After 6 days, multicellular breast cancer spheroids were generated from a heterogeneous population of tumor cells in special culture conditions.

We used non‐adherent 96 well plates for spheroid formation among the heterogeneous population of tumor cells. The spheroids formed in the well after 6 days, as seen in Figure 2. The spheroids were ready to be treated with the extract at this point.

### 3.3 Cytotoxic effects of frankincense extract against monolayer and multicellular breast cancer spheroids

**FIGURE 3:**
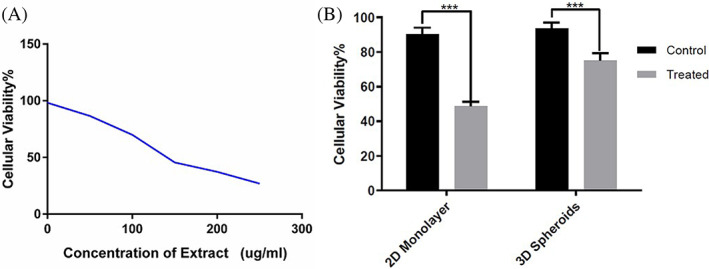
Breast cancer cell viability in 2D monolayer and 3D multicellular spheroids under different concentrations of frankincense extract. (A) MTT assay results quantify the viability of cells under different treatment conditions. Untreated cells were used as the control. Exposing a heterogeneous population of tumor cells to extract resulted in a significant decrease in cell viability in a dose‐dependent manner. The IC50 value was 105 μg/ml by MTT assay. (B) Breast cancer cells in 2D and 3D cultures were treated with frankincense (105 μg/ml) for 48 h. After treatment, 50% of the cells in 2D culture and only 20% of the cells located in the spheroids are affected (***p < .05).

A diverse population of tumor cells was treated with different extract concentrations for 48 h to assess the growth inhibitory activity of frankincense extract on breast cancer cells. Cell viability was determined using the MTT method. The preliminary findings showed that the cytotoxic effects were more qualitative and statistically analyzable after 48 h. As a result, only a dose‐dependent MTT assay was performed. The vitality of primary tumor cells was significantly reduced dose‐dependent after exposure to extract (*p* < .05, Figure 3A). The IC50 value was defined as the concentration of the extract that resulted in a 50% reduction in cell viability compared to the negative control. MTT experiment revealed that the IC50 value was 105 μg/ml. For all subsequent mechanistic experiments, IC50 doses were chosen.

MTT assay was used to measure the antiproliferative properties of frankincense extract on spheroids. Cell viability was determined after cells were treated with the extract (105 μg/ml) for 48 h. The results showed that the extract has significant cytotoxic effects on spheroids. Although, breast cancer cells in the spheroids (CSLCs) are more resistant to these impacts than breast cancer cells in 2D cell culture. As shown in Figure 3B, only 20% of the CSLCs are affected at a concentration that 50% of the breast cancer cells in 2D culture are affected. Compared to other anticancer drugs and compounds such as curcumin and paclitaxel,^20^ frankincense extract had significantly stronger cytotoxic effects against multicellular breast cancer spheroids.

### 3.4 Apoptotic effects of Frankincense extract

**FIGURE 4:**
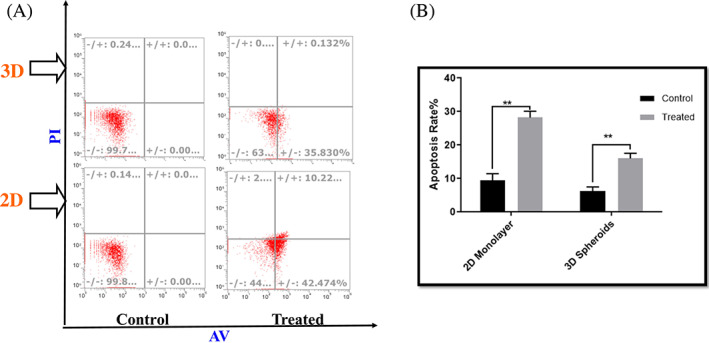
Frankincense regulates apoptosis in breast cancer cells in 2D monolayer and 3D multicellular spheroids. (A) Breast cancer cells in 2D and 3D cultures were treated with frankincense for 48 h. Cell apoptosis was analyzed by flow cytometry (B). After frankincense extract treatment, apoptosis was 28% in 2D conditions, but this level was 16% in spheroids containing cancer stem cells (**p < .001).

The annexin test was utilized to detect the extract's apoptotic effects. The population of tumor cells in two‐dimensional circumstances and the population of CSLCs in spheroids were treated with a concentration of 105 μg/ml extract for this purpose. Figure 4 shows the findings of Annexin V/PI staining for frankincense after 48 h. The extract triggered apoptosis in breast cancer cells in 2D and 3D circumstances. The level of apoptosis was 28% in two‐dimensional conditions with a 105 μg/ml concentration of frankincense and 16% in spheroids containing CSLCs.

### 3.5 Frankincense extract significantly reduces cells migration

**FIGURE 5:**
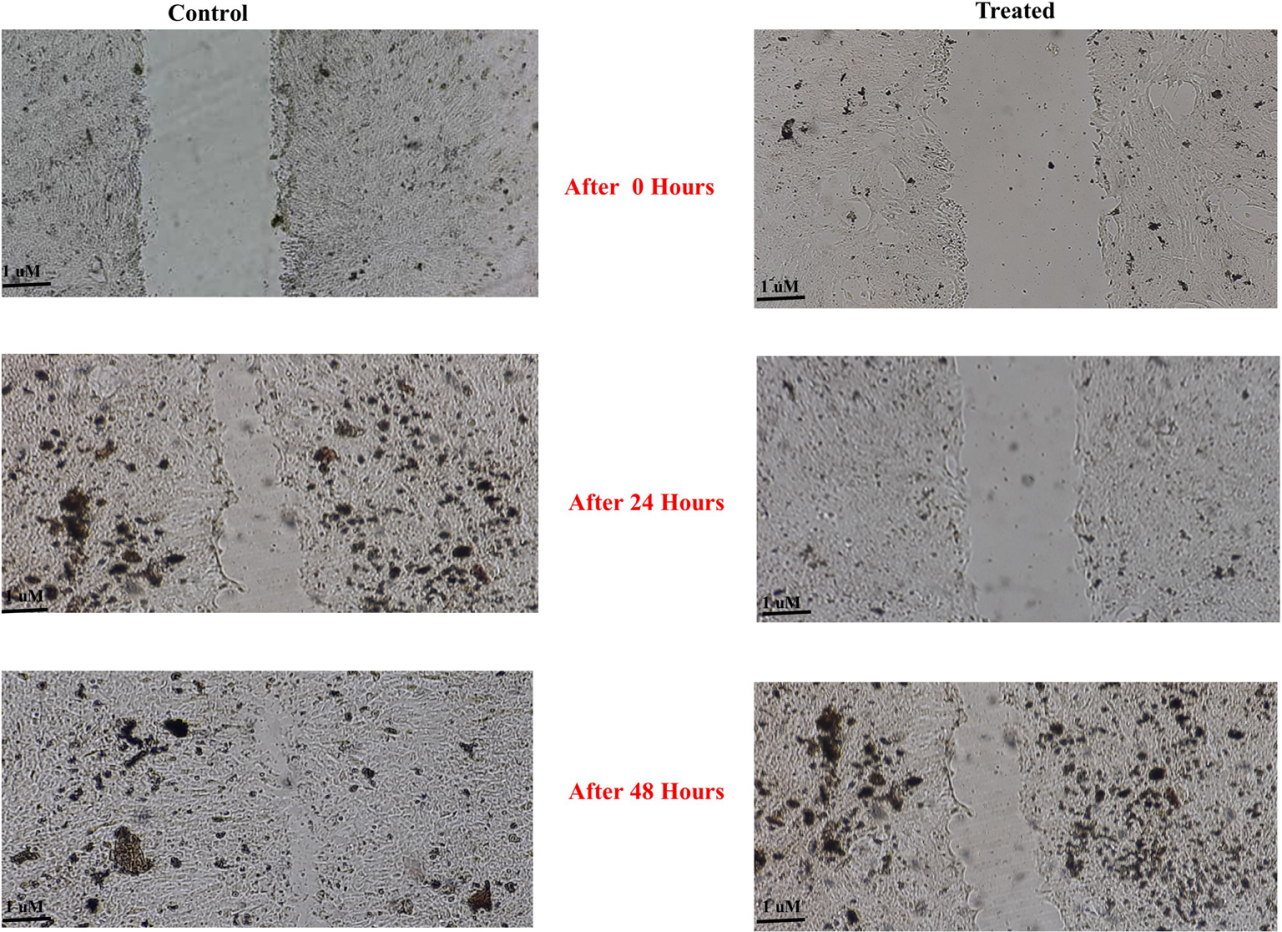
Frankincense suppresses the migration and invasion of a heterogeneous population of tumor cells. The migration ability was compared in treated and control groups based on wound healing.

This stage involved examining the effect of frankincense extract on the migration of a heterogeneous population of tumor cells. Figure 5 depicts the findings of the wound healing assay. Cell migration was extremely rapid in the control group, reaching 40% after only 24 h. After 48 h, there were only faint indications of the wound. In the presence of a 105 μg/ml extract dosage, the motility of the heterogeneous population of tumor cells was dramatically reduced. Despite the extract treatment, the wound did not close entirely after 48 h.

## 4 DISCUSSION

Most investigations on the effects of various antitumor medicines and chemicals on tumor cell lines are conducted utilizing two‐dimensional culture systems. We need to undertake studies on three‐dimensional culture systems and specific cell populations (such as cancer stem cells) to better and more correctly measure these impacts. Relapse and metastasis in cancer patients are linked to a portion of cancer cells with cell stemness. Current cancer treatments are ineffective against these CSCs. Although the fact that CSCs make up a small percentage of total tumor cells, in vitro approaches have been used to enrich these cells.

Cancer cells can form spheroids in an optimal growth culture. Compared to cells cultivated in a 2D monolayer culture, these multicellular spheroids have altered cell surface markers.^21^, ^22^ These spheroids have been employed in several of studies to investigate the in vitro and in vivo features of CSCs, and to assess the inhibitory and cytotoxic activities of anticancer drugs against these cells.^23^, ^24^, ^25^ In the current study, we used tumor cells isolated from a mouse model of TNBC to conduct sphere formation in these cells. The ability of cancer stem cells to form spheres is a critical functional characteristic of these cells. After isolation of methanolic extract of frankincense, the antitumor properties of this extract against cancer stem cells were assessed in the following step. The frankincense extract has cytotoxic and apoptotic effects on cells, according to our findings. However, breast cancer stem cells are more resistant to apoptosis than breast cancer cells.

Many studies have been conducted to see if frankincense has anticancer qualities. The J82 cell line in bladder cancer can be considerably suppressed by frankincense oil and boswellic acid (a main component of Boswellia).^26^ *B. Serrata* extract prevents tumor development in mice by inhibiting pro‐inflammatory cytokine upregulation, according to Huang et al.^27^ Low quantities of Boswellic acids can trigger apoptosis and have potent cytotoxic effects on malignant glioma cells in glioma.^28^ Acetyl‐11‐keto‐beta‐boswellic acid (AKBA) at 10 mg/kg/day has also been found to suppress tumor growth in animals with prostate tumors. It was discovered that AKBA reduces tumor angiogenesis in this kind of malignancy.^29^ Regarding safety issues, past reports have found no major, long‐term, or irreversible side effects associated with *B. Serrata*. As a result, it can be used to treat various of disorders, including cancer, with minimal risk.^30^

The antitumor effects of frankincense have been studied extensively, particularly in breast cancer. The antitumor properties of an alcoholic extract of *B. Serrata* gum resin were studied in vitro and in vivo. It was discovered that the extract causes cell‐specific cytotoxicity in vitro and reduces cell proliferation, angiogenesis, and metastatic rate in vivo.^15^ By decreasing lipoxygenase‐2, *B. Serrata* lowers metastasis and brain tumor growth in patients with breast cancer brain metastases.^14^

The effects of Boswellia sacra essential oil on various human breast cancer cells (T47D, MCF‐7, and MDA‐MB‐231) and normal breast cells (MCF10‐2A) revealed that this essential oil induces tumor cell apoptosis and suppresses tumor aggressiveness. However, normal breast tissue cells were extremely resistant to *B. sacra* oil.^31^ The effects of frankincense extract on murine and human breast cancer and non‐cancer cell lines revealed that at relatively high concentrations, frankincense extract was cytotoxic in MCF‐7. However, this extract was highly effective in blocking invasion in six cancer cells, particularly triple‐negative breast cancer cells (MDA‐MB‐231). As a result, frankincense may effectively prevent breast cancer cell proliferation and metastasis.^32^

These effects on breast cancer stem cells have never been seen before. A highly tumorigenic subpopulation of cells from the breast cancer cell line (T47‐D) was isolated in only one study, and the apoptotic effects of frankincense essential oil on these cells were validated.^33^ Due to many passages and modifications, most breast cancer cell lines have changed their function and genome. Therefore, we decided to make a spheroid out of a heterogeneous population of primary tumor cells. This research utilized a more physiological 3D spheroid culture model, which better simulates the impact of the tumor microenvironment and cell–cell contact on cellular responsiveness to therapy.

Cancer stem cells have been proven resistant to standard chemotherapy treatments in many lab investigations.^34^, ^35^ Several natural substances, such as curcumin,^24^ piperine,^24^ and sulforaphane^36^ have recently been discovered to have anti‐CSCs properties. However, toxicity and poor dose–response limit their application to a large extent. In our work, we proposed frankincense as a new natural substance with anti‐CSCs properties whose clinical applications must be further analyzed.

## 5 CONCLUSIONS

For the first time, the anticancer effects of frankincense extract on breast cancer stem‐like cells were investigated in our study. Frankincense has great anticancer effects on breast cancer stem cells compared to many anticancer medications and chemicals. By focusing on cancer stem cells and establishing a therapy targeting this population, we can overcome metastatic breast cancer in the foreseeable future.

## AUTHOR CONTRIBUTIONS

**Mohammad Kamalabadi Farahani:** Conceptualization (lead); data curation (lead); formal analysis (equal); funding acquisition (lead); investigation (equal); methodology (lead); project administration (lead); resources (lead); software (lead); supervision (lead); validation (lead); visualization (lead); writing – original draft (lead). **Fateme Sadat Bitaraf:** Formal analysis (equal); methodology (equal). **Amir Atashi:** Formal analysis (equal); writing – review and editing (equal). **Zahra Jabbarpour:** Writing – review and editing (equal).

## FUNDING INFORMATION

This study was supported by Grant No. 9886 from Shahroud University of Medical Sciences.

## CONFLICT OF INTEREST

The authors have stated explicitly that there are no conflicts of interest in connection with this article.

## ETHICS STATEMENT

The ethics committee of Shahroud University of medical sciences approved this study for ethics in animal research (registration number: IR.SHMU.REC.1398.109). All studies were carried out in compliance with the ARRIVE guidelines (https://arrive.guidelines.org) for the reporting of animal experiments. All methods were performed in accordance with the relevant guidelines and regulations.

## Data Availability

Data sharing is not applicable to this article as no new data were created or analyzed in this study.
